# Epidemiological Study of Japanese Encephalitis Virus in Vientiane, Lao PDR, in 1990s

**DOI:** 10.1155/2015/235934

**Published:** 2015-01-28

**Authors:** Mika Saito, Douangdao Soukaloun, Khampe Phongsavath, Bounlay Phommasack, Yoshihiro Makino

**Affiliations:** ^1^Department of Microbiology and Oncology, Graduate School of Medicine, University of the Ryukyus, 207 Uehara, Nishihara, Okinawa 903-0215, Japan; ^2^Department of Pediatrics, Mahosot Hospital, Mahosot Road, 01030 Vientiane, Laos; ^3^Department of Pediatrics, Setthathirath Hospital, Boulevard Kamphengmeuang, 01030 Vientiane, Laos; ^4^Department of Communicable Disease Control, Ministry of Health, Si Mouang Road, 01030 Vientiane, Laos; ^5^Shimane Environment & Health Public Corporation, 1-4-6, Koshihara, Matsue, Shimane 690-0012, Japan

## Abstract

Phylogenetic analysis of Japanese encephalitis virus (JEV) was conducted using core-premembrane and envelope gene sequence data of two strains from Vientiane, Lao People's Democratic Republic, in 1993 and five from Okinawa, Japan, in 2002 and 2003, and previously published strains. The two Vientiane strains designated as LaVS56 and LaVS145 belonged to genotype 1 (G1) and the same subcluster of G1 as Australian strain in 2000, Thai strains in 1982–1985 and 2004-2005, and Vietnamese strain in 2005, but were distinct from the subcluster of recently distributing G1 strains widely in Asia including Okinawan strains and recent Lao strain in 2009. These clusters with own distinct distributions indicated involvements of different mechanisms and routes of spreading viruses and clarified that Australian G1 strain is from Southeast Asia, not from East Asia. Both Vientiane strains were antigenically close to P19-Br (G1, isolate, Thailand), but distinct from Nakayama (G3, prototype strain, Japan), Beijing-1 (G3, laboratory strain, China), and JaGAr#01 (G3, laboratory strain, Japan), demonstrated by cross-neutralization tests using polyclonal antisera. These results together with seroepidemiologic study conducted in Vientiane strongly suggest that diversified JEV cocirculated there in early 1990s.

## 1. Introduction

Japanese encephalitis (JE) is a feared disease with a high mortality rate and grave sequelae in the form of neurological and mental impairments [1]. The pathogen responsible for the disease, Japanese encephalitis virus (JEV), belongs to the* Flavivirus *genus of the family Flaviviridae, a single-stranded positive-sense RNA virus, which includes emerging and reemerging pathogens such as West Nile virus and dengue viruses [2, 3]. The genome of JEV is approximately 11,000 bases in length and encodes 3 structural proteins (C: capsid, PrM: premembrane, and E: envelope proteins) and 7 nonstructural proteins (NS1, NS2A, NS2B, NS3, NS4A, NS4B, and NS5). JEV is transmitted to humans by vector mosquitoes from vertebrates, with pigs usually acting as the principal viremic host, and birds, particularly ardeid species, serving as maintenance reservoirs in the wild [2, 4].* Culex tritaeniorhynchus* is the most efficient vector and breeds in bodies of stagnant water such as paddy fields [5].

Five genotypes of JEV based on structure protein sequences, with geographically distinct distributions, have been described [6–8]. Genotype 2 (G2) has been isolated in Southern Thailand, Malaysia, Papua New Guinea (PNG), and Northern Australia, with one strain recognized in the Republic of Korea (ROK) prior to 1951 [9]. Genotype 3 (G3) has been widely distributed throughout Asia. Genotype 4 (G4) has only been isolated in Indonesia between 1980 and 1981 [7]. Only one Singapore strain from 1951 had been recognized as genotype 5 (G5), but it recently reemerged in China and ROK after more than 50 years [10–12].

Before the 1990s, genotype 1 (G1) was distributed in a limited area, from Northern Thailand to Cambodia. It has since moved into Vietnam, China, ROK, Japan, Taiwan, and even Australia, mostly via the so-called genotype-shift phenomenon, whereby a preexisting genotype disappears and is supplanted by another genotype [13–17]. This phenomenon was suggested not to be caused by the intensity of selection [12, 18]. Migratory birds, winds capable of carrying vector insects, travel, and transport are believed to have important roles in the spread of emerging genotypes to new territories; however, the mechanisms involved remain unclear [4].

Lao PDR is a landlocked country situated on the Indochinese peninsula. Geographically, the Annamite Range and the Mekong River lie on its borders, separating the country from Thailand and Vietnam. Only a little information about JE in Lao PDR is available. Okuno [19] first described 34 seasonal encephalitis cases possibly caused by JEV in 1974. Several seroepidemiological studies were conducted on JE in Lao PDR in the 1990s and 2000s [20–25]. An endemic pattern was discovered by studies in which prevalence rates of JE antibodies increased gradually with age in residents and changed seasonally in slaughtered swine in the 1990s [21]. Mackenzie et al. [4] speculated that the low numbers of reported cases were due to a lack of accurate indications and the absence of good surveillance. According to recent reports, an epidemic of JE is occurring [26], with about 50 cases diagnosed serologically between 2001 and 2008 at the largest hospitals in Vientiane [27]. Vaccination programme now includes JE vaccine using SA14-14-2 strains in Lao PDR.

The genetics of JEV is well studied in Lao's neighbors Thailand and Vietnam, which had epidemics of JE during the 1990s, with G1 and G3 as the major genotypes, respectively, but not in Lao PDR. After a case of the genotype-shift phenomenon, namely, G3 to G1 in Vietnam, the major subclusters of G1 Vietnamese and Thai strains were different [28]. Recently, the complete sequence of a JEV isolate from a patient in 2009 in Lao PDR was reported [29]; however, antigenic and genetic analyses of JEV were not described.

In the present study, we sequenced the C/PrM and E protein regions of two JEV isolates from swine sera collected in Vientiane, Lao PDR, in 1993, and analyzed those of isolates phylogenetically by comparison with sequenced G1 Okinawan strains and previously published sequence data of strains from different regions and periods, as well as antigenically by cross-neutralization testing and seroepidemiologically. The possible routes of JEV spread and coexistence of diverse forms of JEV strains in Vientiane, Lao PDR, are discussed.

## 2. Material and Methods

### 2.1. Cell Culture

The mosquito* Aedes albopictus* clone C6/36 cell line was grown at 28°C with Eagle's minimum essential medium (MEM) supplemented with seven nonessential amino acids and 8% heat-inactivated fetal bovine serum (FBS). Baby hamster kidney-21 (BHK-21) and African green monkey kidney (Vero) cell clones were grown at 37°C in 8% FBS-Eagle's MEM as growth medium. All cell clones were maintained with 2% FBS-Eagle's MEM as maintenance medium in a 5% CO_2_ atmosphere when the virus isolation and neutralization test were being conducted.

### 2.2. Collection of Serum Samples

A total of 641 swine serum samples were collected monthly in 1993 and 1994, from domestically reared pigs at a private slaughterhouse in Vientiane Municipality of Lao PDR [21].

Human serum samples from 45 viral encephalitis-suspected patients were collected at acute and convalescent phases in 1994 at two of the largest hospitals in Vientiane. Samples from 8 patients confirmed to have JE in Chiang Mai, Thailand, in 1991 were kindly supplied by Associate Professor Sittisombut, Chiang Mai University. Samples from 2 confirmed cases of JE in Okinawa in 1991 were also used [30]. A total of 278 serum samples from children under 13 years old were collected at the Ministry of Health kindergarten and Paxai Primary School, both in the Sisattanak District, located in an urban area of Vientiane, from January to March (dry season), 1994.

Antisera against 5 strains of JEV, Nakayama (a prototype and vaccine strain, Tokyo, Japan, human brain, 1935, G3), Beijing-1 (a laboratory and vaccine strain, Beijing, China, human brain, 1949, G3), P19-Br (an isolate, Chiang Mai, Thailand, human brain, 1982, G1), LaVS56 (an isolate, Vientiane, Lao PDR, swine sera, 1993, G1), and LaVS145 (an isolate, Vientiane, Lao PDR, swine sera, 1993, G1), were prepared in BALB/c mice as described previously [31]. Before serologic testing, sera were inactivated at 56°C for 30 minutes.

### 2.3. Isolation and Identification of Viruses

A total of 196 among 641 swine serum samples that tested negative for JE antibodies in an IgG ELISA [21, 32] were tested for the virus. The virus isolation and identification procedure were as described previously [15, 33].

### 2.4. Sequences

Two hundred and forty nucleotides of the core-premembrane (C/PrM) gene region and 1500 nucleotides of the envelope (E) gene region of two Vientiane JEV isolates and the E region of five Okinawan isolates, Oki 431S, Oki 128S, Oki 568S, Oki 585S, and Oki 589S, were sequenced (Table 1). Viral RNA was extracted from a JEV-infected C6/36 cell culture by using an RNeasy Mini Kit (Qiagen, Hilden, Germany) or QIAamp Viral RNA Mini Kit (Qiagen) according to the manufacturer's instructions. RT-PCR was performed using the QIAGEN OneStep RT-PCR Kit (Qiagen) also according to the manufacturer's instructions. RT-PCR products were purified using a QIAamp MinElute column (Qiagen) or MinElute Gel Extraction Kit (Qiagen). The products were then sequenced with a BigDye Terminator v.1.1 or v.3.1 cycle sequence kit (Applied Biosystems) and further purified using a DyeEx 2.0 Spin Kit (Qiagen). The sequencing reactions were analyzed on an ABI PRISM 310 and 3100 DNA sequencer (Applied Biosystems). The primers referred to previous publications [14, 34].

### 2.5. Nucleic Acid Sequence Analysis

Details of all the JEV strains used in the phylogenetic analysis are listed in Table 1. Multiple alignments and the phylogenetic analysis were performed by the neighbor-joining (NJ) method using Clustal-X [35]. The bootstrap probabilities of each node were calculated using 1000 replicates. All of the phylogenetic trees were drawn using the NJplot program [36]. For phylogeographic analysis, a map JEV G1 was drawn with published data based on sequences of the E protein, except for Malaysian isolates for the C/PrM region [37].

### 2.6. Serologic Test

For serological analysis of human sera from acute encephalitis cases, six JEV strains (Nakayama, Beijing-1, JaGAr#01, P19-Br, LaVS56, and LaVS145), four prototype strains of dengue virus type 1 (Hawaiian), 2 (New Guinea B), 3 (H-87), and 4 (H-241), and a strain of WNV (Eg-101) were used for fifty percent focus-reduction neutralization tests, as described previously [15].

For the antigenic analysis using mouse antisera, cross-neutralization tests against Nakayama, Beijing-1, JaGAr#01, P19-Br, LaVS56, and LaVS145 were conducted.

For the seroepidemiological analysis using sera obtained from children, neutralization tests against Nakayama, Beijing-1, and LaVS145 were employed. Sera positive for neutralizing antibodies against any of the three strains were used for the comparison of neutralization reactivity. Neutralizing antibody titers against three strains were plotted in correlation graphs. Pearson's correlation coefficient was used for paired values. A statistical analysis of Pearson's correlation coefficient was conducted using the* t*-test.

## 3. Results

### 3.1. Phylogenetic Analysis

**Figure 1:**
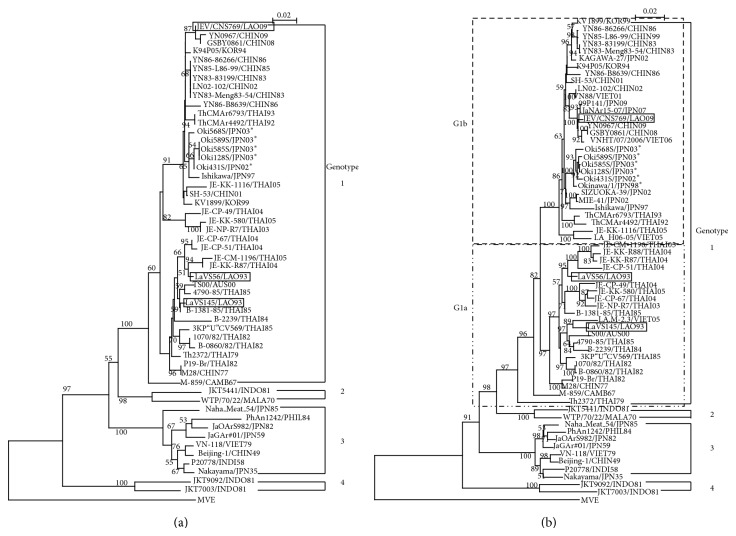
Phylogenetic analysis of JEV strains, with the Murray Valley encephalitis virus strain MVE 1–51 (GenBank accession number NC-000943) as an outgroup. (a) Tree based on a 240-nucleotide sequence of the core-premembrane gene region. (b) Tree based on a 1500-nucleotide sequence of the envelope gene region. Vientiane isolates are indicated in the box, and Okinawan isolates are indicated as ^*^. G1a subcluster and G1b subcluster of JEV genotype 1, indicated in the dot line box, according to Nitpattana and others (2008).

Two strains of virus, LaVS56 and LaVS145, were recovered from serum samples of slaughtered swine collected on May 27 and October 4, 1993, respectively, in Vientiane, Lao PDR. Both strains were identified as belonging to genotype 1 (G1) of JEV from nucleotide sequences of the C/PrM and E gene regions (Table 1 and Figure 1).

Phylogenetic trees of C/PrM and E have similar construction, without contradiction between genotypes and clusters. Percent homologies of the two Vientiane strains are 98.3% at the nucleotide level and 98.8% at the amino acid level in C/PrM and 96.6% at the nucleotide level and 99.0% at the amino acid level in the E region. All of the 6 Okinawan G1 isolates (5 in this study and one published) created a unique Okinawan cluster based on the E region.

According to Nitatpattana et al. [38], LaVS56 and LaVS145 belonged to a subcluster, G1a, corresponding to Lineage 1, the oldest lineage among G1 according to a study by Gao et al. [39] (Figure 1(b)). Further analysis based on the E region revealed LaVS145 to be closely related to strain LA.M-2.3, isolated in Vietnam in 2005, to TS00, isolated in Northern Australia in 2000, and to Thai strains, isolated between 1982 and 1985. LaVS56 was also closely related to Thai isolates from 2004 and 2005. LaVS56 and LaVS145 belonged to different genetic groups within G1a. The G1b subcluster, by contrast, comprised isolates from East and Southeast Asia [28], including a recent Lao strain from 2009, and Okinawan G1 strains.

**Figure 2:**
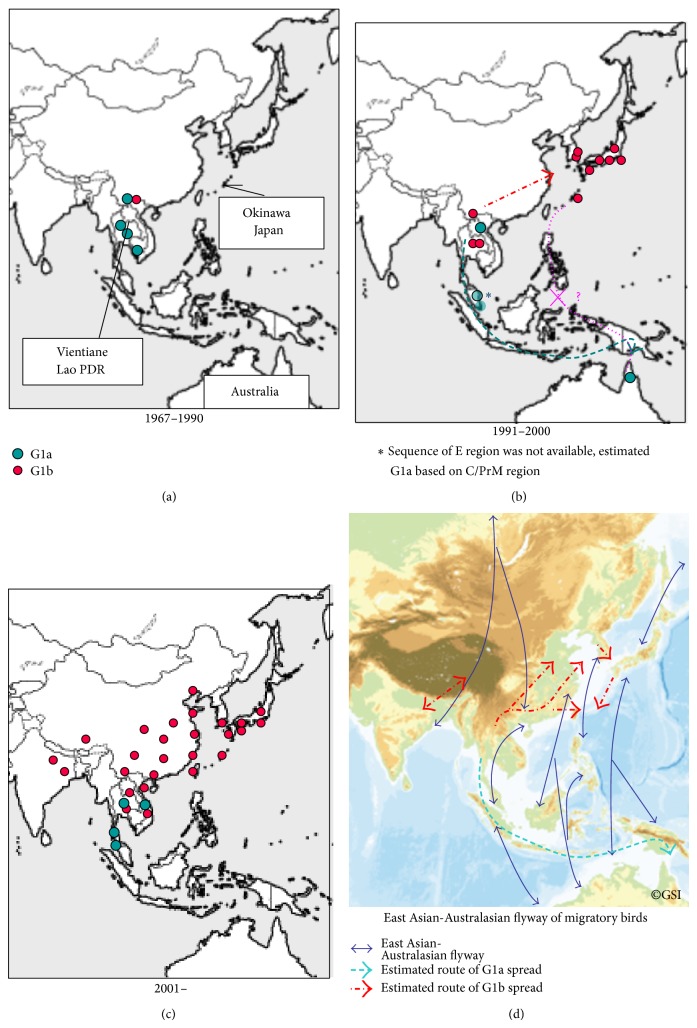
The distribution of subclusters of genotype 1 JEV in each time period, 1967–1990, 1991–2000, and 2001–. The blue dots represent G1a and red dots G1b represent isolates analyzed in this study and published strains. The lower right panel shows an altitude map with the East Asian-Australasian flyways of migration cited from http://www.ozcoasts.gov.au/indicators/shorebird_counts.jsp and estimated migration routes of G1a and G1b.

The distribution of subclusters of G1, namely, G1a and G1b, is shown in Figure 2. The G1 strains between 1967 and 1990 were distributed in a limited area on the Indochinese peninsula, but since then, G1a and G1b have been introduced into geographically distinct regions. Before 1990, G1a was distributed from Yunnan, China, to Northern Thailand and Cambodia, while G1b was recognized only in Yunnan. During the 1990s, the distribution of each cluster expanded, with G1a spreading into Lao PDR, Malaysia, and Australia, while G1b spread into Thailand and to the east into ROK and Japan, including Okinawa. The situation of G1 during the 1990s in inland and coastal China remained unknown. After 2000, G1a was found limited in Northeast and Southern Thailand and Vietnam, while G1b was isolated from widespread Asian regions, such as Vietnam, Northeast Thailand, Lao PDR, Northern India, Yunnan, Tibet, inland and coastal China, ROK, Taiwan, and Japan including Okinawa [15–17, 28, 38–44].

This map probably indicates that the Australian G1 isolate, G1a, in 2000 originated from Southeast Asia, such as from Cambodia, Yunnan, Thailand, Lao PDR, and Malaysia through the Sunda Islands, and not from East Asia, such as China and Japan through Okinawa, Taiwan, and the Philippines, where G1a strains had not been isolated. This map supports the hypothesis that Indochina, the southernmost Asian region, was the source of JEV transmission to the Asian countries for both G1a and G1b [39].

### 3.2. Antigenic Characterization of Two Vientiane Isolates

**Figure 3:**
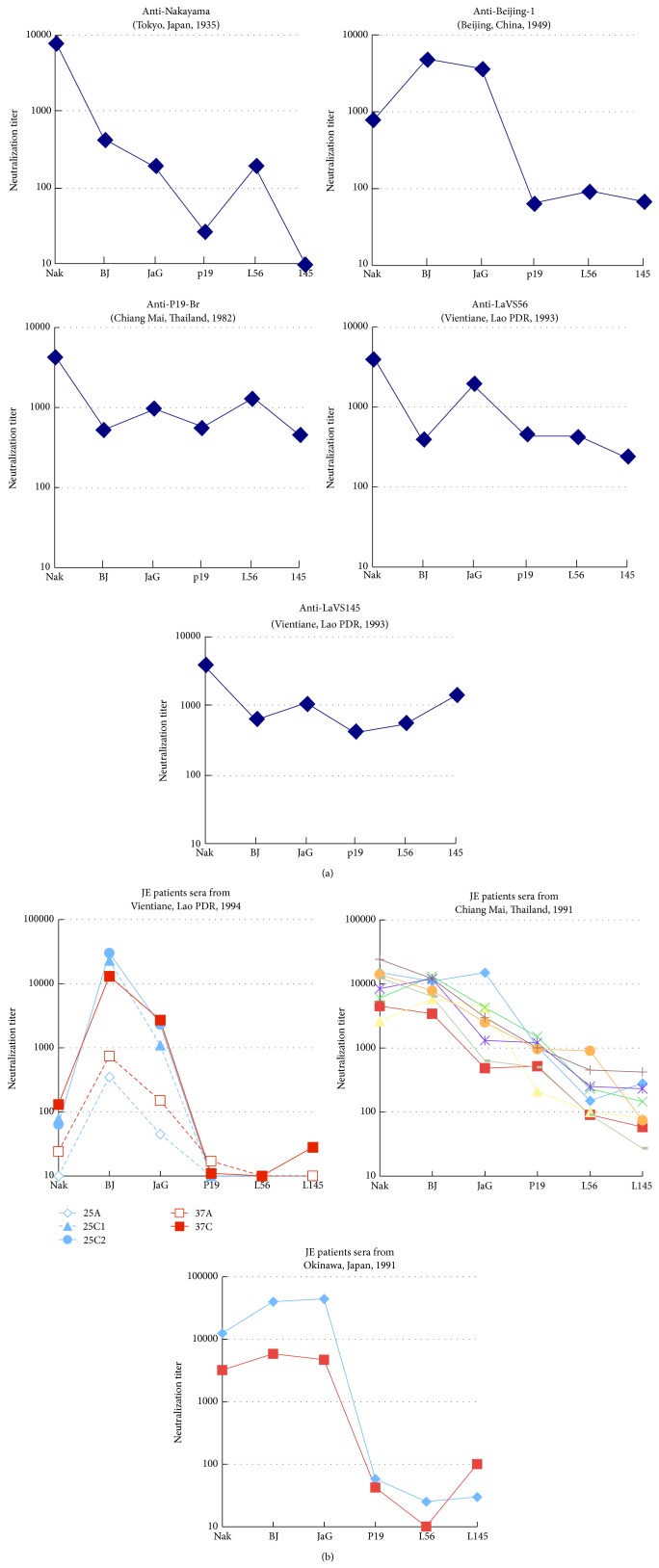
(a) Cross-neutralization testing on 6 JEV strains using polyclonal mouse antisera inoculated with different JEV strains: Nakayama, Beijing-1, P19-Br, LaVS56, and LaVS145. Vertical line: neutralizing antibody titers against JEV strains: Nakayama (Nak), Beijing-1 (BJ), JaGAr#01 (JaG), P19-Br (P19), LaVS56 (L56), and LaVS145 (L145) in a log_10_ scale. (b) The reactivity of sera from JE patients of different regions: two cases of JE (A: acute sera, C: convalescent sera) from Vientiane, Lao PDR, in 1994; eight cases from Chiang Mai, Thailand, in 1991; and two cases from Okinawa, Japan, in 1991. Vertical line: neutralizing antibody titers against JEV strains as panel (a).

Both of the Vientiane isolates in 1993 were antigenically characterized by cross-neutralization testing using antisera against 5 JEV strains: two strains representative of established antigenic subtypes, namely, Nakayama (prototype, vaccine and laboratory strain, G3) and Beijing-1 (vaccine and laboratory strain, G3), a previously characterized strain: P19-Br (Chiang Mai isolate, G1) [31, 45], and two Vientiane isolates: LaVS56 and LaVS145 (Figure 3(a)). Nakayama and Beijing-1 were antigenically distinguishable from each other. The Nakayama and Beijing-1 antisera neutralized the homologous strains, while the titers to the heterologous strains were about 10- to 1000-fold lower. The reactivity of the antisera against P19-Br, LaVS56, and LaVS145 behaved in a similar way, indicating that both Vientiane strains belong to the same antigenic group as P19-Br, as previously described for the ThCAr6793 subtype by Ali and Igarashi [31]. Namely, these sera of the P19-Br group neutralized Nakayama, Beijing-1, JaGAr#01 (the same antigenic group as Beijing-1), P19-Br, LaVS56, and LaVS145 as homologous strains, suggesting the presence of a broader cross-neutralizing epitope in the antigenic domain.

### 3.3. Serologic Characteristics of JE Patients in Three Regions

Forty-five cases clinically diagnosed as viral encephalitis were admitted to the largest two hospitals, Mahosot and Sethathirath Hospitals, in Vientiane, Lao PDR in 1994. Among them, two patients, code numbers 25 and 37, were serologically diagnosed with JE, and another two patients were diagnosed with dengue encephalopathy by neutralization tests and IgM-captured ELISA for JE and dengue (data not shown). A total of 41 cases had an unknown etiology. This result together with previous seroepidemiological data [20–22] suggests that Lao PDR was a JE-endemic country early in the 1990s. Interestingly, the sera of both JE patients showed a significant increase of more than 4-fold between acute and convalescent sera in terms of the neutralizing antibody titers against Beijing-1 (vaccine strain, G3), JaGAr#01 (laboratory strain, G3), and Nakayama (vaccine strain, G3), but not against P19-Br (G1, Chiang Mai, Thailand), LaVS145 (G1, Vientiane, Lao PDR), or LaVS56 (G1, Vientiane, Lao PDR) (Figure 3(b)). In addition, convalescent-phase sera from eight JE patients in Chiang Mai, Thailand, in 1991 and two JE patients in Okinawa, Japan, in 1991 showed unique neutralization patterns in each area (Figure 3(b)). The serum samples from Chiang Mai reacted with the Chiang Mai, Vientiane, and laboratory strains. The samples from Okinawa had similar neutralization patterns to those from Vientiane and the antisera for Beijing-1 that rarely reacted with Vientiane and Chiang Mai strains. On the other hand, the samples from Chiang Mai had similar patterns to the antisera for P19-Br, LaVS56, and LaVS145. JE patients in Vientiane seemed to be infected with the Beijing-1 serogroup, not the P19-Br serogroup.

### 3.4. Neutralization Reactivity in Healthy Children in Vientiane

**Figure 4:**
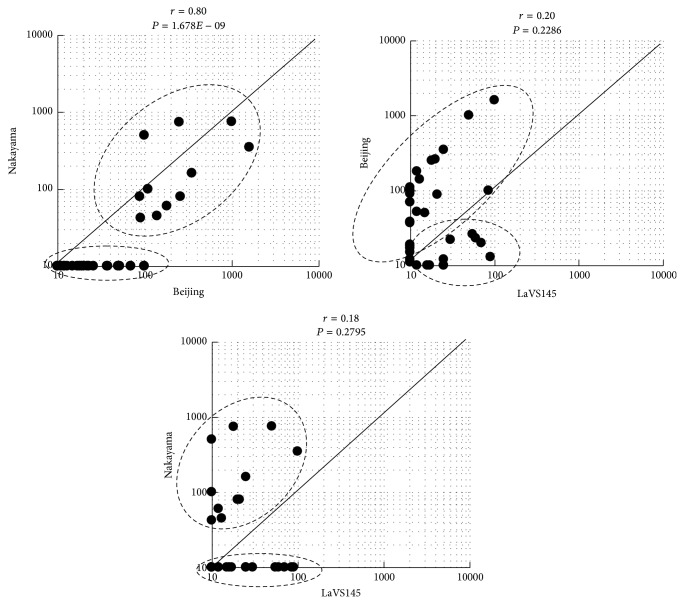
Correlation graphs of neutralizing antibody titers against three strains of Japanese encephalitis virus in children in Vientiane in 1994, between JEV strains. The *x*- and *y*-axes show neutralization titers against each strain. The correlation coefficient is described as an *r* value. The result of a *t*-test for each correlation coefficient is shown as a *P* value.

Since vaccination programs in Lao PDR did not include the JE vaccine in 1990s, the presence of the JE antibody in children may reflect the natural transmission of JEV. Among 278 serum samples taken from children under 13 years old in Vientiane in 1994, 38 had antibodies against any of the 3 JEV strains, Nakayama, Beijing-1, and LaVS145 (Figure 4). The samples tended to be divided into two groups: those that neutralized Nakayama or Beijing-1 strongly and those that neutralized LaVS145 strongly. The coefficients of the relationships between Beijing-1 and LaVS145 and between Nakayama and LaVS145 were low and not significant (*P* > 0.1).

## 4. Discussion

To our knowledge, this is the report on the earliest JEV isolates from Vientiane in Lao PDR. Phylogeographic analysis of JEV G1 strains including Vientiane and Okinawan strain elucidated different clusters, G1a and G1b, with distinct distribution, evidenced that there are different mechanisms and routes to spread JEV G1 into new area, and proposed that the Australian G1a isolate originated from Thailand and Lao PDR region, Southeast Asia, but not from East Asia though Okinawa, Taiwan, probably through Philippines. The recent expansion of G1 in Asian countries has mainly involved G1b. Our data also support the view of Gao et al. [39] that Indochina, the southernmost Asian region, was the source of JEV transmission to the Asian countries.

Although both distributions correlate with East Asian-Australasian flyways of migratory birds [13, 39, 46, 47], the direction of spreading clusters is different; G1a is from north to south, while G1b is from west to east. Mackenzie et al. [48] hypothesized that JEV reached Australasia by island hopping across the eastern Indonesia archipelago by birds, particularly ardeid birds, establishing the bird-mosquito and pig-mosquito transmission cycles on each island as it moved. Ritchie and Rochester [49] calculated mosquitoes that could carry virus from PNG to islands in northern Australia during cyclonic weather patterns. On the other hand, Nabeshima et al. [28] and Morita [50] suggested that jet wind and westerly wind carrying small insects might be involved in G1 spread to East Asia beside migratory birds. Further study on the present situation of JEV in Indonesia and the Philippines will give us the clear map for the routes of expansion.

It was also believed that JEV was carried northward to and within Japan [51], since yearly seasonal activity of JEV had been started from Okinawa as southern-most area of Japan. The recent appearance of G1b in Taiwan has been about 600 km southwest from Okinawa Island, approximately 10 years after its first appearance in China, ROK, and Japan [40]. The Taiwanese G1 strains are related to strains in coastal China more than to those in Japan. Phylogenetic analysis suggested that JEV has been introduced into Okinawa from the north, such as ROK and other parts of Japan, and a serosurvey of migratory birds in Okinawa [52] suggests that it has been possibly carried by winter visitors.

The antigenic and genetic variation of JEV has been studied extensively. Nakayama and Beijing-1, both belonging to the G3 subgroup, are known to differ in antigenicity [4]. Antigenic variation was also reported within G1. The two Vientiane strains were of the same antigenic serogroup as P19-Br and ThCMAr6793, according to Ali and Igarashi [31], most commonly observed in G1 by cross-neutralization testing, characterized by antisera that neutralize a wide range of JEV strains including members of not only homologous antigenic groups but also distinct antigenic groups, while antisera of Nakayama and Beijing-1 neutralize specifically homologous serogroups. The immune status of G3 in humans and pigs might have exerted pressure for genotype shift because of this difference.

Interestingly, sera from JE patients in Vientiane showed Beijing-1-type seroreactivity, similar to the antiserum of Beijing-1 and JE patients in Okinawa, Japan, in 1991, but different from the antisera for LaVS56, LaVS145, and P19-Br, and JE patients in Chiang Mai, Thailand, exhibited P19-Br-type seroreactivity. Only G3 had been isolated in Okinawa until 1992. These results indicate that the patients in Vientiane were infected by the Beijing-1 serogroup. Therefore, there were at least two different antigenic groups, Beijing-1-type and P19-Br-type, of JEV in Vientiane. Furthermore, the seroepidemiological study of healthy children in Vientiane strengthened the notion that there was a distinct antigenic group of JEV.

As no JEV G1 strains were reported to belong to the Beijing-1 serogroup, G1 and G3 may have coexisted in Vientiane in the early 1990s. In addition, the Vientiane strains, LaVS56 and LaVS145, belong to genetically different subgroups of the same G1a cluster. It is evident that antigenically and genetically diverse forms of JEV cocirculated in a limited area and over a limited period of time. In addition, many live pigs were imported for food into Lao PDR from Vietnam in those days, where G3 had been the major genotype. This might be the possible route and mechanism of expansion.

It is strongly suggested that report of JE cases was underestimated because of absence of good surveillance and accurate laboratory diagnosis [4, 26]. In this study period of the early 1990s, no epidemic outbreak of JE was observed, and many suspected viral encephalitis cases were with unknown etiology in Lao PDR. This together with seroepidemiological studies [20–22] revealed an endemic but not epidemic status of JE in Lao PDR. The reason why JE epidemic did not occur in Vientiane is unknown, although serologic study in swine showed that JEV activity was not very high, which might suggest low density of vector mosquitoes there [21]. Coexistence of diverse form of JEV might be enabled by very local transmission cycle as a result of this situation.

At present, no specific treatment is available for JE. Work on the production of vaccines using Nakayama, Beijing-1, and SA14-14-2, all members of G3, is ongoing. Although epidemiological evidence of the efficiency of JE vaccine was well reported [53, 54], given the recent expansion of G1 in Asia, careful assessments of the efficiency, safety, and validity of ongoing vaccines using G3 strains including SA14-14-2 used in Lao PDR are continuously needed. As JE is easily influenced by environmental and socioeconomic changes, risk assessments from multilateral approaches not only in Asia and Oceania but also Europe and America are required to control the disease.

## 5. Conclusion

Japanese encephalitis remains still one of the public health threats in Asia including Lao PDR. In this study, the subclusters of G1, G1a and G1b, with their own distinct distributions indicate the involvement of different mechanisms and routes of spread and propose that the Australian G1a originated from Southeast Asia through Sunda Island, not from East Asia. Our study also supports the hypothesis that Indochina was the source of JEV transmission to the Asian countries for the both G1a and G1b. In addition, the results together with antigenic and seroepidemiological studies strongly suggest that diverse antigenicity of JEV cocirculated, with endemic pattern in Lao PDR, 1990s.

## Figures and Tables

**Table 1:** JEV isolates analyzed in this study.

Strain	Year	Location	Source	Genotype	Accession number	
					C/PrM	E
Nakayama	1935	Tokyo, Japan	Human brain	3	U03694	S75726
Beijing-1	1949	Beijing, China	Human brain	3	L48961	L48961
P20778	1958	Vellore, India	Human brain	3	AF080251	AF080251
JaGAr#01	1959	Gunma, Japan	*C. tritaeniorhynchus *	3	D00961	AF069076
M-859	1967	Cambodia	*C. gelidus *	1	D00984	U70410
WTP/70/22	1970	Kuala Lumpur, Malaysia	Mosquito	2	D00998	U70421
M28	1977	Yunnan, China	*C. pseudovishnui *	1	JF706279	JF706279
Th2372	1979	Thailand	Human brain	1	D76424	U70401
VN-118	1979	Ho Chi Minh, Vietnam	*C. fatigans *	3	D00975	U70420
JKT7003	1981	Indonesia	Mosquito	4	L42161	U70408
JKT9092	1981	Indonesia	Mosquito	4	L42158	U70409
JKT5441	1981	Indonesia	Mosquito	2	L42164	U70406
P19-Br	1982	Chiang Mai, Thailand	Human brain	1	D76427	JEU70416
B-0860/82	1982	Thailand	Pig	1	GQ902058	GQ902058
1070/82	1982	Thailand	Human	1	GQ902059	GQ902059
JaOArS982	1982	Japan	Mosquito pool	3	M18370	M18370
YN83-Meng83-54	1983	Yunnan, China	*Lasiohelea taiwana Shiraki *	1	DQ404062	DQ404130
YN83-83199	1983	Yunnan, China	*Culex sp. *	1	DQ404063	DQ404131
PhAn 1242	1984	Santo Cristo, Philippines	Swine blood	3	D00982	U70417
B-2239	1984	Chiang Mai, Thailand	Swine blood	1	D00993	U70391
B-1381-85	1985	Thailand	Pig	1	GQ902061	GQ902061
3KP“U”CV569	1985	Thailand	Mosquito	1	GQ902060	GQ902060
4790-85	1985	Thailand	Human	1	GQ902062	GQ902062
YN85-L86-99	1985	Yunnan, China	*Culex sp. *	1	DQ404064	DQ404132
Naha Meat 54	1985	Okinawa, Japan	Swine blood	3	D85958	DQ355367^*^
YN86-B8639	1986	Yunnan, China	*C. tritaeniorhynchus *	1	DQ404065	DQ404133
YN86-86266	1986	Yunnan, China	NA	1	DQ404066	DQ404134
ThCMAr4492	1992	Chiang Mai, Thailand	mosquito	1	D45360	D45362
ThCMAr6793	1993	Chiang Mai, Thailand	*C. tritaeniorhynchus *	1	D45361	D45363
LaVS56	1993	Vientiane, Lao PDR	Swine serum	1	GQ850124^*^	GU815346^*^
LaVS145	1993	Vientiane, Lao PDR	Swine serum	1	GQ850123^*^	GU815345^*^
K94P05	1994	Korea	*C. tritaeniorhynchus *	1	AF045551	AF045551
Ishikawa	1997	Ishikawa, Japan	Swine mononuclear cell	1	AB051292	AB051292
Okinawa/1	1998	Okinawa, Japan	Wild boar	1	NA	AB306941
KV1899	1999	Korea	NA	1	AY316157	AY316157
TS00	2000	Australia	Swine serum	1	AF289814	EF434785
SH-53	2001	Shanghai, China	*C. tritaeniorhynchus *	1	AY555746	AY555757
VN88	2001	Vietnam	Swine blood	1	NA	AY376464
LN02-102	2002	Liaoning, China	*C. modestus *	1	DQ404018	DQ404085
Oki 431S	2002	Okinawa, Japan	Swine serum	1	DQ355361	DQ355369^*^
MIE-41	2002	Mie, Japan	Swine serum	1	NA	AB112709
SIZUOKA-39	2002	Shizuoka, Japan	Swine serum	1	NA	AB112704
KAGAWA-27	2002	Kagawa, Japan	Swine serum	1	NA	AB112707
Oki 128S	2003	Okinawa, Japan	Swine serum	1	DQ355362	DQ355368^*^
Oki 568S	2003	Okinawa, Japan	Swine serum	1	DQ355363	DQ355370^*^
Oki 585S	2003	Okinawa, Japan	Swine serum	1	DQ355364	DQ355371^*^
Oki 589S	2003	Okinawa, Japan	Swine serum	1	DQ355365	DQ355372^*^
JE-NP-R7	2003	Thailand	Swine blood	1	DQ084216	DQ084228
JE-CP-49	2004	Chumphon, Thailand	Swine blood	1	DQ084223	DQ087974
JE-CP-51	2004	Chumphon, Thailand	Swine blood	1	DQ084225	DQ087973
JE-CP-67	2004	Chumphon, Thailand	Swine blood	1	DQ084222	DQ087972
JE-KK-R87	2004	Khon Kaen, Thailand	Swine blood	1	DQ084218	DQ111788
JE-KK-R88	2004	Khon Kaen, Thailand	Swine blood	1	DQ084219	DQ111786
LA-H06-05	2005	Vietnam	mosquitoes	1	NA	FJ185153
LA.M-2.3	2005	Vietnam	*C. pseudovishnui *	1	NA	JN574432
JE-CM-1196	2005	Chiang Mai, Thailand	Swine	1	DQ356483	DQ238602
JE-KK-580	2005	Khon Kaen, Thailand	Swine	1	DQ356481	DQ238600
JE-KK-1116	2005	Khon Kaen, Thailand	Swine	1	DQ388999	DQ343290
VNHT/07/2006	2006	Ha Tai, Vietnam	*C. tritaeniorhynchus *	1	NA	AB728498
JaNAr17-07	2007	Nagasaki, Japan	mosquitoes	1	NA	FJ185149
GSBY0861	2008	Gansu, China	*C. tritaeniorhynchus *	1	JN381833	JN381833
09P141	2009	Oita, Japan	Swine	1	NA	GU108335
YN0967	2009	Yunnan, China	*C. tritaeniorhynchus *	1	JF706268	JF706268
JEV/CNS769	2009	Vientiane, Laos	Human brain	1	KC196115	KC196115

NA: not available, ^*^sequenced in this study.

## References

[1] Johnson R. T., Burke D. S., Elwell M. (1985). Japanese encephalitis: immunocytochemical studies of viral antigen and inflammatory cells in fatal cases. *Annals of Neurology*.

[2] Monath T. P., Heinz H. X., Fields B. N., Knipe D. M., Howley P. M. (1996). Flaviviruses. *Fields Virology*.

[3] Mackenzie J. S., Gubler D. J., Petersen L. R. (2004). Emerging flaviviruses: the spread and resurgence of Japanese encephalitis, West Nile and dengue viruses. *Nature Medicine*.

[4] Mackenzie J. S., Williams D. T., Smith D. W., Tabor E. (2007). Japanese encephalitis virus: the geographic distribution, incidence, and spread of a virus with a propensity to emerge in new areas. *Emerging Virus in Human Population*.

[5] Doi R., Shirasaka A., Sasa M., Oya A. (1977). Studies on the susceptibility of three species of mosquitoes to Japanese encephalitis virus. *Journal of Medical Entomology*.

[6] Chen W.-R., Tesh R. B., Rico-Hesse R. (1990). Genetic variation of Japanese encephalitis virus in nature. *Journal of General Virology*.

[7] Chen W.-R., Rico-Hesse R., Tesh R. B. (1992). A new genotype of Japanese encephalitis virus from Indonesia. *The American Journal of Tropical Medicine and Hygiene*.

[8] Solomon T., Ni H., Beasley D. W. C., Ekkelenkamp M., Cardosa M. J., Barrett A. D. T. (2003). Origin and evolution of Japanese encephalitis virus in Southeast Asia. *Journal of Virology*.

[9] Schuh A. J., Li L., Tesh R. B., Innis B. L., Barrett A. D. T. (2010). Genetic characterization of early isolates of Japanese encephalitis virus: genotype II has been circulating since at least 1951. *Journal of General Virology*.

[10] Uchil P. D., Satchidanandam V. (2001). Phylogenetic analysis of Japanese encephalitis virus: envelope gene based analysis reveals a fifth genotype, geographic clustering, and multiple introductions of the virus into the Indian subcontinent. *The American Journal of Tropical Medicine and Hygiene*.

[11] Li M.-H., Fu S.-H., Chen W.-X. (2011). Genotype v japanese encephalitis virus is emerging. *PLoS Neglected Tropical Diseases*.

[12] Mohammed M. A. F., Galbraith S. E., Radford A. D. (2011). Molecular phylogenetic and evolutionary analyses of Muar strain of Japanese encephalitis virus reveal it is the missing fifth genotype. *Infection, Genetics and Evolution*.

[13] Nga P. T., del Carmen Parquet M., Cuong V. D. (2004). Shift in Japanese encephalitis virus (JEV) genotype circulating in Northern Vietnam: implications for frequent introductions of JEV from Southeast Asia to East Asia. *Journal of General Virology*.

[14] Ma S.-P., Yoshida Y., Makino Y., Tadano M., Ono T., Ogawa M. (2003). A major genotype of Japanese encephalitis virus currently circulating in Japan. *The American Journal of Tropical Medicine and Hygine*.

[15] Saito M., Taira K., Itokazu K., Mori N. (2007). Recent change of the antigenicity and genotype of Japanese encephalitis viruses distributed on Okinawa Island, Japan. *The American Journal of Tropical Medicine and Hygiene*.

[16] Wang H. Y., Takasaki T., Fu S. H. (2007). Molecular epidemiological analysis of Japanese encephalitis virus in China. *Journal of General Virology*.

[17] Chung Y.-J., Nam J.-H., Ban S.-J., Cho H.-W. (1996). Antigenic and genetic analysis of Japanese encephalitis viruses isolated from Korea. *American Journal of Tropical Medicine and Hygiene*.

[18] Tang W.-F., Ogawa M., Eshita Y., Aono H., Makino Y. (2010). Molecular evolution of Japanese encephalitis virus isolates from swine in Oita, Japan during 1980–2009. *Infection, Genetics and Evolution*.

[19] Okuno T. (1978). An epidemiological review of Japanese encephalitis. *World Health Statistics Quarterly*.

[20] Fukunaga T., Phommasack B., Bounlu K. (1993). Epidemiological situation of dengue infection in Lao P.D.R. *Tropical Medicine*.

[21] Makino Y., Saito M., Phommasack B. (1995). Arbovirus infections in pilot areas in Laos. *Tropical Medicine*.

[22] Vongxay P., Makino Y., Kanemura K., Saito M., Fukunaga T. (1995). Seroepidemiological study of arbovirus infection in Khammouane Province, Lao PDR. *Ryukyu Medical Journal*.

[23] Vallée J., Dubot-Pérès A., Ounaphom P., Sayavong C., Bryant J. E., Gonzalez J.-P. (2009). Spatial distribution and risk factors of dengue and Japanese encephalitis virus infection in urban settings: the case of Vientiane, Lao PDR. *Tropical Medicine & International Health*.

[24] Hiscox A., Winter C. H., Vongphrachanh P. (2010). Serological investigations of flavivirus prevalence in Khammouane Province, Lao People’s Democratic Republic, 2007-2008. *The American Journal of Tropical Medicine and Hygine*.

[25] Conlan J. V., Vongxay K., Jarman R. G. (2012). Serologic study of pig-associated viral zoonoses in Laos. *The American Journal of Tropical Medicine and Hygiene*.

[26] Erlanger T. E., Weiss S., Keiser J., Utzinger J., Wiedenmayer K. (2009). Past, present, and future of Japanese encephalitis. *Emerging Infectious Diseases*.

[27] Moore C. E., Blacksell S. D., Taojaikong T. (2012). A prospective assessment of the accuracy of commercial IgM ELISAs in diagnosis of Japanese encephalitis virus infections in patients with suspected central nervous system infections in Laos. *The American Journal of Tropical Medicine and Hygiene*.

[28] Nabeshima T., Loan H. T. K., Inoue S. (2009). Evidence of frequent introductions of Japanese encephalitis virus from south-east Asia and continental east Asia to Japan. *Journal of General Virology*.

[29] Aubry F., Vongsouvath M., Nougairède A. (2013). Complete genome of a genotype I Japanese encephalitis virus isolated from a patient with encephalitis in Vientiane, Lao PDR. *Genome Announcements*.

[30] Saito M., Sunagawa T., Makino Y. (1999). Three Japanese encephalitis cases in Okinawa, Japan, 1991. *Southeast Asian Journal of Tropical Medicine and Public Health*.

[31] Ali A., Igarashi A. (1997). Antigenic and genetic variations among Japanese encephalitis virus strains belonging to genotype 1. *Microbiology and Immunology*.

[32] Bundo K., Morita K., Igarashi A. (1982). Enzyme-linked immunosorbent assay (ELISA) on Japanese encephalitis virus. III. Assay on antibody titers in swine sera. *Tropical Medicine*.

[33] Igarashi A., Chiowanich P., Leechanachai P., Supawadee J. (1983). Virological and epidemiological studies on encephalitis in Chiang Mai Area, Thailand, in the year of 1982. III. Virus isolation from clinical materials. *Tropical Medicine*.

[34] Morita K., Tadano M., Nakaji S. (2001). Locus of a virus neutralization epitope on the Japanese encephalitis virus envelope protein determined by use of long PCR-based region-specific random mutagenesis. *Virology*.

[35] Thompson J. D., Gibson T. J., Plewniak F., Jeanmougin F., Higgins D. G. (1997). The CLUSTAL X windows interface: flexible strategies for multiple sequence alignment aided by quality analysis tools. *Nucleic Acids Research*.

[36] Perrière G., Gouy M. (1996). WWW-query: an on-line retrieval system for biological sequence banks. *Biochimie*.

[37] Tsuchie H., Oda K., Vythilingam I. (1997). Genotypes of Japanese encephalitis virus isolated in three states in Malaysia. *The American Journal of Tropical Medicine and Hygiene*.

[38] Nitatpattana N., Dubot-Pérès A., Gouilh M. A. (2008). Change in Japanese encephalitis virus distribution, Thailand. *Emerging Infectious Diseases*.

[39] Gao X., Liu H., Wang H., Fu S., Guo Z., Liang G. (2013). Southernmost Asia is the source of Japanese encephalitis virus (genotype 1) diversity from which the viruses disperse and evolve throughout Asia. *PLoS Neglected Tropical Diseases*.

[40] Huang J.-H., Lin T.-H., Teng H.-J. (2010). Molecular epidemiology of Japanese encephalitis virus, Taiwan. *Emerging Infectious Diseases*.

[41] Sarkar A., Taraphdar D., Mukhopadhyay S. K., Chakrabarti S., Chatterjee S. (2012). Molecular evidence for the occurrence of Japanese encephalitis virus genotype I and III infection associated with acute encephalitis in patients of West Bengal, India, 2010. *Virology Journal*.

[42] Fulmali P. V., Sapkal G. N., Athawale S., Gore M. M., Mishra A. C., Bondre V. P. (2011). Introduction of Japanese encephalitis virus genotypei, India. *Emerging Infectious Diseases*.

[43] Chen S. P. (2012). Molecular phylogenetic and evolutionary analysis of Japanese encephalitis virus in China. *Epidemiology and Infection*.

[44] Li Y. X., Li M. H., Fu S. H. (2011). Japanese encephalitis, Tibet, China. *Emerging Infectious Diseases*.

[45] Hasegawa H., Yoshida M., Kobayashi Y., Fujita S. (1995). Antigenic analysis of Japanese encephalitis viruses in Asia by using monoclonal antibodies. *Vaccine*.

[46] World press org http://www.eaaflyway.net/.

[47] Jelsoft Enterprises Ltd http://www.flutrackers.com/forum/showthread.php?t=147.

[48] Mackenzie J. S., Johansen C. A., Ritchie S. A., van den Hurk A. F., Hall R. A. (2002). Japanese encephalitis as an emerging virus: the emergence and spread of Japanese encephalitis virus in Australasia. *Current Topics in Microbiology and Immunology*.

[49] Ritchie S. A., Rochester W. (2001). Wind-blown mosquitoes and introduction of Japanese encephalitis into Australia. *Emerging Infectious Diseases*.

[50] Morita K. (2009). Molecular epidemiology of Japanese encephalitis in East Asia. *Vaccine*.

[51] Rosen L. (1986). Natural history of Japanese encephalitis virus. *Annual Review of Microbiology*.

[52] Saito M., Ito T., Amano Y., Růžek D. (2011). Trials for risk assessment of Japanese encephalitis based on serologic surveys of wild animals. *Flavivirus encephalitis*.

[53] Hoke C. H., Nisalak A., Sangawhipa N. (1988). Protection against Japanese encephalitis by inactivated vaccines. *The New England Journal of Medicine*.

[54] Bista M. B., Banerjee M. K., Shin S. H. (2001). Efficacy of single-dose SA 14-14-2 vaccine against Japanese encephalitis: a case control study. *The Lancet*.

